# Association between nausea and vomiting during pregnancy and adverse pregnancy outcomes: findings from the nuMoM2b study

**DOI:** 10.1007/s00404-025-08176-3

**Published:** 2025-09-03

**Authors:** Ya-Ling Hsieh, Chia-Jung Chiang, Tsung Yu

**Affiliations:** 1 Nursing Department, Chi Mei Medical Center, Tainan, Taiwan; 2 Department of Obstetrics and Gynecology, National Cheng Kung University Hospital, College of Medicine, National Cheng Kung University, Tainan, Taiwan; 3 Department of Public Health, College of Medicine, National Cheng Kung University, NCKU Hospital Outpatient Building Floor 8, 138 Sheng-Li Road, North District, Tainan, 704302 Taiwan

**Keywords:** Nausea and vomiting of pregnancy, Hyperemesis gravidarum, Gestational weight gain, Adverse pregnancy outcomes

## Abstract

**Purpose:**

Nausea and vomiting of pregnancy (NVP), including its severe form hyperemesis gravidarum (HG), have been linked to various perinatal outcomes, though findings remain inconsistent. This study aimed to examine the association between NVP severity and adverse pregnancy outcomes and to evaluate whether gestational weight gain (GWG) mediates these relationships.

**Methods:**

We analyzed data from 8396 nulliparous women enrolled in the U.S. nuMoM2b cohort. NVP severity was measured using the Pregnancy-Unique Quantification of Emesis (PUQE) score across three prenatal visits and categorized as none, one, and ≥2 visits of medium-to-severe NVP. Perinatal outcomes included birth weight, gestational age, preterm delivery, small for gestational age (SGA), and low birth weight (LBW). GWG adequacy was assessed per Institute of Medicine guidelines. Multivariable regression models were used, adjusting for sociodemographic and clinical covariates.

**Results:**

Overall, 81.6% of women reported no visit with medium-to-severe NVP, 16.2% with one such visit, and 2.3% with two or three such visits. One visit with medium-to-severe NVP was associated with a modest reduction in birth weight (−41.4 g; 95% CI: −72.6, −10.2). Inadequate GWG—regardless of NVP status—was consistently associated with shorter gestation (−0.53 weeks), lower birth weight (−261.3 g), and increased risks of preterm birth, LBW, and SGA (ORs 1.66–2.75).

**Conclusion:**

NVP severity alone showed limited impact on short-term pregnancy outcomes. However, inadequate GWG emerged as a key modifiable risk factor. These findings underscore the importance of nutritional support and symptom management during pregnancy, particularly for women with moderate-to-severe NVP. Long-term outcomes warrant further investigation.

**Supplementary Information:**

The online version contains supplementary material available at 10.1007/s00404-025-08176-3.

## What does this study add to the clinical work?

**Table:** 

This study demonstrates that inadequate gestational weight gain, rather than the severity of NVP, is the key contributor to adverse perinatal outcomes such as preterm birth, low birth weight, and small for gestational age. Clinically, this highlights the importance of monitoring and supporting adequate maternal weight gain in women with NVP to optimize pregnancy outcomes.	

## Introduction

Nausea and vomiting of pregnancy (NVP), commonly referred to as “morning sickness,” are frequent symptoms during early pregnancy, while its severe form, hyperemesis gravidarum (HG), can lead to serious complications such as weight loss, ketonemia, and electrolyte imbalance [1]. Numerous studies, including several systematic reviews, have investigated the associations between NVP or HG and adverse pregnancy outcomes. However, the findings have been inconsistent, particularly in differentiating the effects of NVP versus HG. For instance, reviews by Veenendaal et al., Jansen et al., and Moberg et al. reported increased risks of preterm delivery, low birth weight (LBW), and small for gestational age (SGA) in pregnancies complicated by HG [2–4]. Reviews by Koren et al. and Moberg et al., however, suggested that NVP may be associated with a reduced risk of preterm birth and LBW [4, 5]. These patterns suggest that milder NVP may not adversely affect outcomes, and in some cases might be associated with a “protective effect,” whereas HG—representing the severe end of the spectrum—is more often associated with adverse maternal and child outcomes through mechanisms such as weight loss and nutritional deficiency.

There are a variety of factors that influence the occurrence and severity of NVP and HG. The strongest risk factors include prior history of NVP/HG, family history and genetic predisposition to the altered hormonal factors, e.g., growth differentiation factor 15 (GDF15). Other factors—such as fetal sex, plurality, maternal age, gravidity, race/ethnicity, socioeconomic status, body mass index (BMI), smoking status, comorbidities, Helicobacter pylori infection—contribute more modestly to risk [6–9]. Many of these variables may act as confounders in the observed relationships between NVP/HG and adverse pregnancy outcomes [10]. Therefore, careful consideration and adjustment for these covariates are essential in studies aiming to clarify these associations.

In addition to potential confounding, the pathway linking NVP/HG to adverse outcomes may involve gestational weight gain (GWG). It is often hypothesized that NVP or HG contributes to inadequate maternal weight gain during pregnancy, which in turn increases the risk of adverse outcomes [1, 11, 12]. Despite this plausible mechanism, relatively few studies have directly examined GWG as a mediator. Notably, a 2006 study by Dodds et al. found that mothers with HG who gained less than 7 kg during pregnancy had significantly higher risks of preterm birth, LBW, and SGA, whereas those who achieved a weight gain of 7 kg or more had outcomes comparable to women without HG [13]. This suggests a potential mediating role of GWG in the relationship between NVP/HG and pregnancy outcomes, though this finding has yet to be consistently replicated across diverse populations.

Given the inconsistencies and limitations of previous research, we conducted an analysis to examine the association between NVP, including HG, and adverse pregnancy outcomes using data from a large U.S. cohort study, the Nulliparous Pregnancy Outcomes Study: Monitoring Mothers-to-Be (nuMoM2b). In particular, our analysis accounted for the severity of NVP, maternal characteristics, and GWG.

## Materials and methods

### Study population

The nuMoM2b study [14], launched in 2009 by the Eunice Kennedy Shriver National Institute of Child Health and Human Development, was designed to identify factors associated with adverse pregnancy outcomes among nulliparous women. Participants were recruited from eight academic medical centers (Case Western Reserve University, Columbia University, Indiana University, University of Pittsburgh, Northwestern University, University of California at Irvine, University of Pennsylvania, and University of Utah), with institutional review board approval obtained at each site.

Eligible participants were nulliparous women with a viable singleton gestation between 6 + 0 and 13 + 6 weeks, confirmed during first-trimester screening at affiliated obstetric clinics. Inclusion required an intent to deliver at a participating hospital and no history of pregnancy reaching ≥20 weeks of gestation.

Data were collected across three standardized study visits: Visit 1 (6 + 0–13 + 6 weeks), Visit 2 (16 + 0–21 + 6 weeks), and Visit 3 (22 + 0–29 + 6 weeks). For this study, we utilized standardized anthropometric measures (height and weight) and data from structured interviews conducted at Visit 1, which captured information on socio-demographics, reproductive and medical history, physical activity, dietary intake, tobacco and alcohol use, and symptoms of depression (Edinburgh Postnatal Depression Scale) and anxiety (State-Trait Anxiety Inventory-Trait Subscale). NVP was assessed at all three visits.

### Exposure variables

NVP was assessed using the Pregnancy-Unique Quantification of Emesis (PUQE) questionnaire, which was developed to quantify the severity of NVP [15]. It consists of three items assessing the duration of nausea, and the frequency of vomiting and retching, each rated on a 5-point scale. The total PUQE score thus ranges from 3 to 15, with scores of 4–6 indicating mild, 7–12 indicating medium, and ≥13 indicating severe NVP. In this study, participants were classified into three groups: those with no visits reporting medium-to-severe NVP, those with one such visit, and those with two or three such visits.

### Outcome variables

We examined the association between the severity of NVP and various perinatal outcomes, including gestational age (continuous), birth weight (continuous), infant sex, gestational diabetes, gestational hypertension/preeclampsia, stillbirth (defined as fetal death at or after 20 weeks of gestation), Apgar scores below 7 at 1 and 5 min, preterm delivery (before 37 weeks of gestation), LBW (<2500 g), SGA (birth weight below the 10th percentile for gestational age and sex), cesarean section, indicated labor (delivery following induction or cesarean section), and admission to a Neonatal Intensive Care Unit (NICU) or acute care setting.

We assessed the impact of NVP severity—categorized as none, one, and two to three visits of medium-to-severe NVP—on these outcomes. Additionally, we stratified the analyses by whether GWG met the minimum recommended levels based on pre-pregnancy BMI, according to the Institute of Medicine guidelines [16].

### Statistical analysis

We examined the associations between NVP and perinatal outcomes using linear regression for continuous outcomes and logistic regression for binary outcomes. Results are presented as mean differences or odds ratios (ORs) with corresponding 95% confidence intervals (CIs). Based on prior literature, we adjusted for maternal age, BMI, household income (expressed as a percentage of the 2013 federal poverty level), race/ethnicity, educational attainment, marital status, smoking, alcohol consumption, gravidity, history of miscarriage, mental health conditions, pre-gestational diabetes, and chronic hypertension. Missing covariate data were handled using multiple imputation. All analyses were performed using SAS version 9.4 (SAS Institute Inc., Cary, NC, USA).

## Results

The initial dataset comprised 9,289 participants. Of these, 8,396 (90.4%) from the nuMoM2b study who completed the PUQE questionnaire at all three visits were included in the analysis. We compared the characteristics of included and excluded participants (Supplementary Table S1). Excluded participants were less likely to be non-Hispanic White, less educated, more likely to be single, and less likely to use vitamins.

**Fig. 1:**
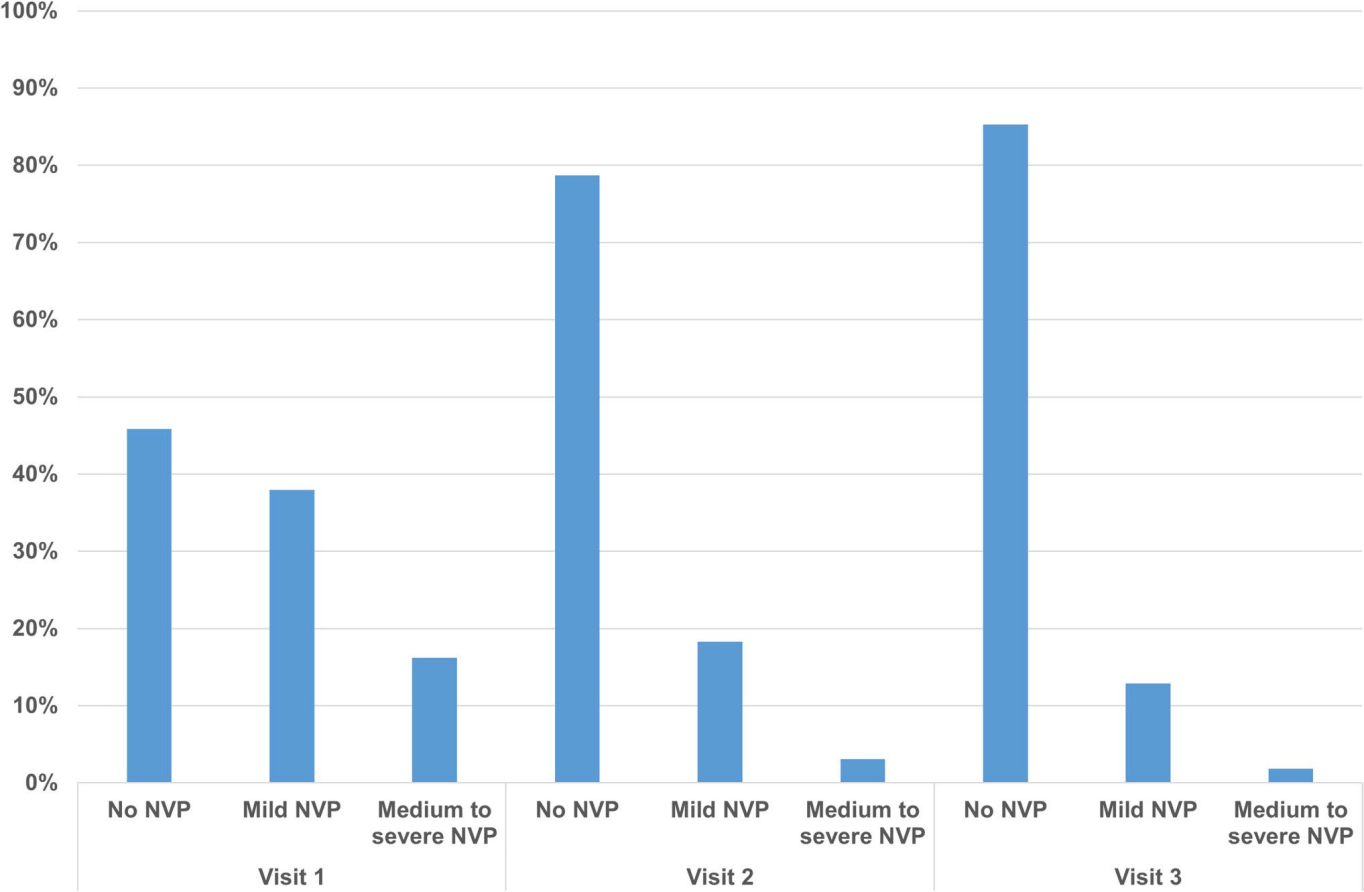
Distribution of nausea and vomiting of pregnancy (NVP) severity across study visits

The prevalence of NVP by severity across study visits is presented in Fig. 1. At Visit 1, 45.8% of participants reported no NVP, 38.0% reported mild NVP, and 16.2% reported medium-to-severe NVP. At Visit 2, the corresponding proportions were 78.7%, 18.3%, and 3.1%, respectively. At Visit 3, the corresponding proportions were 85.3%, 12.9%, and 1.8%, respectively. We also summarized the distribution of participants by the presence of medium-to-severe NVP across study visits (Supplementary Table S2). Among the 8396 participants, 81.6% (6849) had no visits with medium-to-severe NVP, 16.2% (1356) had one such visit, and 2.3% (191) had two to three such visits.

Table 1 presents the distribution of demographic and clinical characteristics among 8396 participants in the nuMoM2b study, stratified by NVP severity: none, one, and two to three visits of medium-to-severe NVP. Participants with greater NVP severity were generally younger, had lower income, and lower educational attainment. For instance, the mean maternal age declined with increasing NVP severity (27.3 years in the first group, 25.8 in the second group, and 25.2 in third group; *p* < 0.001). Hispanic women were more likely to report medium-to-severe NVP, and participants with greater NVP severity were less likely to be married.

**Table 1:** Distribution of demographic and clinical characteristics among study participants

	No medium-to-severe NVP (N = 6849)		1 visit with medium-to-severe NVP (N = 1356)		≥ 2 visits with medium-to-severe NVP (N = 191)		
Variable	M or n	SD or %	M or n	SD or %	M or n	SD or %	*P-value*
Numerical variable							
Maternal age (year)	27.3	5.6	25.8	5.3	25.2	4.8	< 0.001
Body mass index	26.2	6.1	26.8	7.0	28.0	7.3	< 0.001
Income as % of FPL	456.0	310.1	359.0	287.1	247.2	233.5	< 0.001
Energy intake (kcal/d)	1689.0	879.5	1788.8	1148.0	1929.5	1387.0	< 0.001
AHEI-2010	55.7	12.6	53.2	11.9	52.2	12.3	< 0.001
EPDS score	5.4	4.0	6.9	4.6	8.2	5.0	< 0.001
STAI-T score	33.3	8.4	35.4	9.2	37.8	9.6	< 0.001
Categorical variable							
Race/ethnicity							0.001
Non-Hispanic White	4318	63.1	798	58.9	115	60.2	
Non-Hispanic Black	860	12.6	186	13.7	22	11.5	
Hispanic	1043	15.2	249	18.4	42	22.0	
Asian	289	4.2	43	3.2	2	1.1	
Other	339	5.0	80	5.9	10	5.2	
Education							< 0.001
Less than HS graduate	499	7.3	114	8.4	22	11.5	
HS graduate or GED	733	10.7	198	14.6	23	12.0	
Some college	1253	18.3	300	22.1	56	29.3	
Assoc/Tech degree	641	9.4	190	14.0	32	16.8	
Completed college	1998	29.2	322	23.8	38	19.9	
Degree work beyond college	1723	25.2	232	17.1	20	10.5	
Marital status							0.183
Single	2545	37.2	529	39.0	84	44.0	
Married	4223	61.7	807	59.5	105	55.0	
Other	79	1.2	20	1.5	2	1.1	
Met physical activity guidelines							< 0.001
No	2485	36.3	498	36.7	74	38.7	
Yes	2462	36.0	332	24.5	42	22.0	
Smoking prior to pregnancy							0.035
No	5658	82.6	1116	82.3	144	75.4	
Yes	1189	17.4	239	17.6	47	24.6	
Drinking prior to pregnancy							< 0.001
No	1361	19.9	349	25.7	52	27.2	
Yes	4630	67.6	719	53.0	89	46.6	
Use of vitamin							0.725
No	773	11.3	161	11.9	24	12.6	
Yes	6076	88.7	1195	88.1	167	87.4	
Gravidity							0.112
1	5133	75.0	1027	75.7	130	68.1	
2	1283	18.7	245	18.1	41	21.5	
>= 3	433	6.3	84	6.2	20	10.5	
History of miscarriage							0.027
No	5789	84.5	1136	83.8	148	77.5	
Yes	1060	15.5	220	16.2	43	22.5	
Mental health conditions							< 0.001
No	5568	81.3	1040	76.7	139	72.8	
Yes	1159	16.9	286	21.1	46	24.1	
Pregestational diabetes							0.127
No	6663	97.3	1328	97.9	185	96.9	
Yes	107	1.6	12	0.9	4	2.1	
Chronic hypertension							0.292
No	6554	95.7	1289	95.1	176	92.2	
Yes	168	2.5	34	2.5	8	4.2	

*AHEI-2010* Alternative Healthy Eating Index 2010, *Assoc/Tech degree* Associate/Technology degree, *NVP* nausea and vomiting of pregnancy, *EPDS* Edinburgh Postnatal Depression Scale, *FPL* federal poverty level, *GED* General Educational Development, *HS* high school, *M* mean, *SD* standard deviation, *STAI-T* State-Trait Anxiety Inventory-Trait Subscale

Women with more severe NVP had higher energy intake, lower dietary quality, and were less likely to meet physical activity guidelines or report alcohol use prior to pregnancy. Higher NVP severity was also associated with increased gravidity, a greater likelihood of previous miscarriage, and a higher prevalence of mental health conditions and chronic hypertension. Additionally, participants with more severe NVP reported significantly higher depression and anxiety scores compared to those with less severe NVP at baseline.

Table 2 presents the comparison of perinatal outcomes across three groups of pregnant individuals: none, one, and two to three visits of medium-to-severe NVP. All analyses were adjusted for relevant covariates. Infants born to mothers with one medium-to-severe NVP had a significantly lower mean birth weight (3253.4 g) compared to those without medium-to-severe NVP (3301.3 g), with a mean difference of −41.4 g (95% CI: −72.6, −10.2). These women also had higher odds of delivering a female infant (OR: 1.19, 95% CI: 1.05–1.34), an SGA infant (OR: 1.27, 95% CI: 1.05–1.55), and experiencing indicated labor (OR: 1.19, 95% CI: 1.05–1.35). Women with two or more visits of medium-to-severe NVP showed similar patterns, including lower mean birth weight (−43.4 g, 95% CI: −120.3, 33.6) and higher odds of indicated labor (OR: 1.36, 95% CI: 1.01–1.85), though most associations were not statistically significant due to limited sample size.

**Table 2:** Perinatal outcomes according to severity of NVP

Outcome	No medium-to-severe NVP (N = 6849)	1 visit with medium-to-severe NVP (N = 1356)	≥ 2 visits with medium-to-severe NVP (N = 191)
Gestational age			
Mean (SD)	38.9 (1.8)	38.8 (1.7)	38.7 (1.7)
Mean difference (95% CI)	0	−0.07 (−0.17, 0.04)	−0.16 (−0.42, 0.10)
Birth weight			
Mean (SD)	3301.3 (532.1)	3253.4 (534.5)	3250.7 (526.4)
Mean difference (95% CI)	0	**−41.4 (−72.6, −10.2)**	−43.4 (−120.3, 33.6)
Female infant			
n (%)	3159 (46.1)	676 (49.9)	88 (46.1)
Odds ratio (95% CI)	1	**1.19 (1.05, 1.34)**	0.99 (0.74, 1.33)
Gestational diabetes			
n (%)	297 (4.3)	56 (4.1)	5 (2.6)
Odds ratio (95% CI)	1	0.95 (0.70, 1.29)	0.57 (0.23, 1.42)
Gestational hypertension or Preeclampsia			
n (%)	1537 (22.4)	277 (20.4)	43 (22.5)
Odds ratio (95% CI)	1	**0.86 (0.74, 1.00)**	0.97 (0.68, 1.40)
Stillbirth			
n (%)	12 (0.2)	7 (0.5)	0 (0)
Odds ratio (95% CI)	1	2.40 (0.91, 6.27)	Not estimable
1-min Apgar score < 7			
n (%)	726 (10.6)	143 (10.6)	23 (12.0)
Odds ratio (95% CI)	1	0.95 (0.78, 1.16)	1.04 (0.66, 1.63)
5-min Apgar score < 7			
n (%)	132 (1.9)	26 (1.9)	3 (1.6)
Odds ratio (95% CI)	1	0.92 (0.60, 1.41)	0.70 (0.22, 2.25)
Preterm delivery			
n (%)	507 (7.4)	106 (7.8)	21 (11.0)
Odds ratio (95% CI)	1	0.98 (0.79, 1.23)	1.35 (0.84, 2.16)
Low birth weight			
n (%)	384 (5.6)	96 (7.1)	9 (4.7)
Odds ratio (95% CI)	1	1.25 (0.99, 1.58)	0.78 (0.39, 1.54)
SGA			
n (%)	593 (8.7)	147 (10.8)	19 (10.0)
Odds ratio (95% CI)	1	**1.27 (1.05, 1.55)**	1.19 (0.73, 1.94)
Cesarean section			
n (%)	1879 (27.4)	336 (24.8)	62 (32.5)
Odds ratio (95% CI)	1	0.90 (0.78, 1.03)	1.31 (0.95, 1.82)
Indicated labor			
n (%)	2265 (33.1)	507 (37.4)	80 (41.9)
Odds ratio (95% CI)	1	**1.19 (1.05, 1.35)**	**1.36 (1.01, 1.85)**
NICU/acute care			
n (%)	923 (13.5)	180 (13.3)	34 (17.8)
Odds ratio (95% CI)	1	0.95 (0.80, 1.14)	1.26 (0.86, 1.85)

All analyses were adjusted for maternal age, body mass index, household income, race/ethnicity, educational attainment, marital status, smoking, alcohol consumption, gravidity, history of miscarriage, mental health conditions, pregestational diabetes, and chronic hypertension

*CI* confidence interval, *NICU* neonatal intensive care unit, *NVP* nausea and vomiting of pregnancy, *SD* standard deviation, *SGA* small for gestational age

Bold values indicate statistically significant associations (p <0.05)

Table 3 presents perinatal outcomes stratified by NVP status (no medium-to-severe NVP vs. ≥1 medium-to-severe NVP) and whether GWG met the Institute of Medicine’s recommendations. In both NVP groups, inadequate GWG was associated with significantly shorter gestational age and lower birth weight. For instance, among women with ≥1 medium-to-severe NVP, inadequate GWG was linked to a 0.53-week reduction in gestational age (95% CI: −0.73, −0.34) and a 261.3 g lower birth weight (95% CI: −319.5, −203.1).

**Table 3:** Perinatal outcomes according to NVP and GWG

Outcome	No medium-to-severe NVP		≥ 1 visit with medium-to-severe NVP	
	GWG meeting recommendations (N = 5581)	GWG not meeting recommendations (N = 1169)	GWG meeting recommendations (N = 1183)	GWG not meeting recommendations (N = 348)
Gestational age				
Mean (SD)	39.0 (1.7)	38.5 (2.1)	38.9 (1.7)	38.4 (2.0)
Mean difference (95% CI)	0	**−0.43 (−0.55, −0.32)**	−0.05 (−0.16, 0.06)	**−0.53 (−0.73, −0.34)**
Birth weight				
Mean (SD)	3345.1 (515.0)	3087.9 (563.4)	3305.1 (520.4)	3060.1 (535.1)
Mean difference (95% CI)	0	**−244.6 (−277.9, −211.2)**	**−36.7 (−69.6, −3.8)**	**−261.3 (−319.5, −203.1)**
Female infant				
n (%)	2561 (45.9)	556 (47.6)	604 (51.1)	155 (44.5)
Odds ratio (95% CI)	1	**1.17 (1.02, 1.33)**	**1.24 (1.09, 1.41)**	1.08 (0.86, 1.36)
Gestational diabetes				
n (%)	218 (3.9)	74 (6.3)	47 (4.0)	14 (4.0)
Odds ratio (95% CI)	1	**1.66 (1.25, 2.20)**	1.02 (0.73, 1.42)	1.07 (0.60, 1.90)
Gestational hypertension or Preeclampsia				
n (%)	1362 (24.4)	160 (13.7)	270 (22.8)	46 (13.2)
Odds ratio (95% CI)	1	**0.47 (0.39, 0.56)**	0.87 (0.75, 1.02)	**0.44 (0.32, 0.62)**
Stillbirth				
n (%)	9 (0.2)	3 (0.3)	4 (0.3)	3 (0.9)
Odds ratio (95% CI)	1	1.48 (0.39, 5.64)	1.67 (0.50, 5.53)	3.94 (0.99, 15.66)
1-min Apgar score < 7				
n (%)	606 (10.9)	111 (9.5)	131 (11.1)	32 (9.2)
Odds ratio (95% CI)	1	0.88 (0.71, 1.09)	0.97 (0.79, 1.18)	0.81 (0.56, 1.19)
5-min Apgar score < 7				
n (%)	105 (1.9)	22 (1.9)	21 (1.8)	7 (2.0)
Odds ratio (95% CI)	1	1.00 (0.63, 1.60)	0.86 (0.53, 1.39)	0.99 (0.45, 2.16)
Preterm delivery				
n (%)	376 (6.7)	125 (10.7)	88 (7.4)	39 (11.2)
Odds ratio (95% CI)	1	**1.72 (1.38, 2.14)**	1.02 (0.80, 1.31)	**1.66 (1.16, 2.37)**
Low birth weight				
n (%)	251 (4.5)	125 (10.7)	65 (5.5)	40 (11.5)
Odds ratio (95% CI)	1	**2.65 (2.11, 3.33)**	1.21 (0.91, 1.61)	**2.75 (1.91, 3.95)**
SGA				
n (%)	408 (7.3)	177 (15.1)	103 (8.7)	61 (17.5)
Odds ratio (95% CI)	1	**2.21 (1.82, 2.68)**	1.20 (0.96, 1.51)	**2.68 (1.98, 3.63)**
Cesarean section				
n (%)	1596 (28.6)	249 (21.3)	329 (27.8)	64 (18.4)
Odds ratio (95% CI)	1	**0.69 (0.59, 0.81)**	0.98 (0.84, 1.13)	**0.60 (0.45, 0.81)**
Indicated labor				
n (%)	1895 (34.0)	334 (28.6)	471 (39.8)	107 (30.8)
Odds ratio (95% CI)	1	**0.82 (0.71, 0.95)**	**1.24 (1.08, 1.41)**	0.90 (0.71, 1.16)
NICU/acute care				
n (%)	759 (13.6)	148 (12.7)	160 (13.5)	51 (14.7)
Odds ratio (95% CI)	1	0.93 (0.77, 1.13)	0.95 (0.78, 1.14)	1.09 (0.80, 1.49)

All analyses were adjusted for maternal age, body mass index, household income, race/ethnicity, educational attainment, marital status, smoking, alcohol consumption, gravidity, history of miscarriage, mental health conditions, pregestational diabetes, and chronic hypertension

*CI* confidence interval, *GWG* gestational weight gain, *NICU* neonatal intensive care unit, *NVP* nausea and vomiting of pregnancy, *SD* standard deviation, *SGA* small for gestational age

Bold values indicate statistically significant associations (p <0.05)

Across both NVP groups, inadequate GWG increased the odds of several adverse outcomes, including preterm delivery (OR: 1.72 and 1.66, respectively), LBW (OR: 2.65 and 2.75), and SGA (OR: 2.21 and 2.68). In contrast, inadequate GWG was associated with lower odds of gestational hypertension or preeclampsia (OR: 0.47 and 0.44), cesarean section (OR: 0.69 and 0.60), and indicated labor (OR: 0.82 and 0.90). The risk of NICU or acute care admission and low Apgar scores did not differ significantly across groups. These findings indicate that regardless of NVP severity, inadequate GWG is consistently associated with increased risks of preterm birth and impaired fetal growth.

## Discussion

In the present study, after extensive covariate adjustment, most outcomes did not differ by NVP severity. One visit with medium-to-severe NVP was linked to a 41.4 g reduction in birth weight vs. no medium-to-severe NVP and was associated with higher odds of having female infant, SGA and indicated labor. Point estimates for birth weight difference, preterm birth and indicated labor trended unfavorably with increasing severity, but only indicated labor reached statistical significance. In the analysis stratified by GWG, it emerged as the dominant driver of risk. Within both the no and ≥1 medium-to-severe NVP groups, failing to meet Institute of Medicine GWG recommendations produced shorter gestation (−0.43 weeks; −0.53 weeks, respectively), markedly lower birth weight (−244.6 g and −261.3 g, respectively) and 1.66–2.75-fold higher odds of preterm delivery, LBW, and SGA. These patterns were consistent regardless of NVP status, underscoring inadequate GWG—not NVP itself—as the key modifiable factor of adverse perinatal outcomes.

One of the key strengths of our analysis was the comprehensive adjustment for a range of potential confounders. Women with more severe symptoms tended to be younger, have lower income and education levels, engage in less healthy behaviors (such as poorer dietary quality and reduced physical activity), and report higher levels of depression and anxiety. Similarly, a study by Roseboom [10] and colleagues examined the characteristics of women with HG and its independent effect on pregnancy outcomes. After adjusting for numerous maternal factors—including age, parity, socioeconomic status, ethnicity, mode of conception, urbanization, substance use, hypertension, diabetes, psychiatric conditions, and fetal sex—they found that the association between HG and adverse outcomes, such as preterm birth and SGA, was largely attributable to these underlying maternal characteristics. Our results align with these findings.

Another strength of this study was the use of a validated instrument—the PUQE questionnaire—to assess the severity of NVP. The nuMoM2b study administered the PUQE questionnaire at three separate study visits, allowing for a more precise evaluation of both the presence and severity of NVP symptoms. In contrast, many previous studies have relied on International Classification of Diseases codes to identify NVP or HG, which may lack consistency in diagnostic criteria and reporting. These inconsistencies likely contribute to the variability observed in associations between NVP/HG and perinatal outcomes across studies.

The present study indicates that, regardless of NVP status, inadequate GWG—rather than NVP itself—is the primary contributor to several adverse pregnancy outcomes, including preterm delivery, LBW, and SGA. Meta-analyses have consistently shown that inadequate GWG is associated with a higher risk of SGA and preterm birth, although it does not appear to be linked to an increased risk of cesarean delivery [17]. In our analysis, inadequate GWG was unexpectedly associated with lower odds of gestational hypertension/preeclampsia and cesarean section, a finding that aligns with results from at least one systematic review [18]. Given that NVP and HG can lead to malnutrition, dehydration, electrolyte imbalances, and unintended weight loss, it is critical to investigate interventions that help prevent inadequate GWG among mothers with NVP/HG. Such interventions may include appropriate medical treatment, nutritional support, and dietary and lifestyle counseling [19].

Although our findings suggest that NVP/HG may not significantly increase the risk of adverse pregnancy outcomes, this interpretation warrants caution. As Roseboom and colleagues noted [10], such conclusions may overlook the long-term effects of fetal programming—where the intrauterine environment shapes lifelong health, independent of fetal growth [20]. The Dutch Hunger Winter (1944–1945) studies offer compelling evidence: early gestational famine exposure increased risks of schizophrenia, depression, atherogenic lipid profiles, and coronary heart disease, while exposure at any stage raised the risk of type 2 diabetes [21]. The physiological stress and nutritional deficits from NVP/HG may mirror maternal starvation. A recent systematic review reported modest increases in neurodevelopmental and mental health disorders—and a possible link to testicular cancer—among offspring of mothers with HG [22], though findings are limited by the low quality and number of available long-term studies, highlighting the need for future research.

This study has several limitations. First, although counting the number of visits with medium-to-severe NVP across three time points provides a more comprehensive assessment of overall NVP severity, it may mask fluctuations in symptoms and their possible time-dependent impact on perinatal outcomes. Second, despite adjusting for a broad range of maternal characteristics and health conditions, the possibility of residual confounding remains. Important factors such as the intensity and treatment of psychiatric symptoms, specific nutritional interventions, and the use of antiemetic medications were not fully accounted for. Lastly, the scope of this study was limited to short-term perinatal outcomes. Given growing evidence supporting the fetal programming hypothesis, future research with long-term follow-up is needed to assess potential impacts on offspring neurodevelopment, metabolic profiles, and overall health trajectories.

To conclude, our results underscore the importance of supporting optimal maternal GWG, particularly among women experiencing moderate to severe NVP, through targeted nutritional guidance, symptom management, and clinical monitoring. While NVP may not directly increase the risk of adverse short-term outcomes, its potential impact on maternal nutrition highlights the need for proactive interventions. Future studies should examine long-term health outcomes in offspring, including potential fetal programming effects associated with maternal undernutrition in the context of NVP/HG.

## Competing interests

The authors declare no competing interests.

## Ethical approval

The nuMoM2b study protocol and procedures were approved by the Institutional Review Boards at all participating sites: Case Western Reserve University, Columbia University, Indiana University, University of Pittsburgh, Northwestern University, University of California at Irvine, University of Pennsylvania, and University of Utah. The Data Coordinating and Analysis Center is RTI International, located in Research Triangle Park, North Carolina.

## Consent to participate

Informed consent was obtained from all participants included in the study.

## Consent to publish

Not applicable.

## Supplementary Information

Below is the link to the electronic supplementary material.

Supplementary file1 (DOCX 21 KB)

## Data Availability

Information about the Nulliparous Pregnancy Outcomes Study: Monitoring Mothers-to-Be (nuMoM2b) dataset used in this research is available on the NICHD DASH website.
