# Supplementary material for: Association between nausea and vomiting during pregnancy and adverse pregnancy outcomes: findings from the nuMoM2b study
Source: Arch Gynecol Obstet. 2025 Sep 3;312(5):1695–704. doi: 10.1007/s00404-025-08176-3 (PMC12589366; doi:10.1007/s00404-025-08176-3)
Supplement: Supplementary file 1 — Supplementary file1 (DOCX 21 KB) [file 404_2025_8176_MOESM1_ESM.docx]

**Association Between Nausea and Vomiting During Pregnancy and Adverse Pregnancy Outcomes: Findings From the nuMoM2b Study**

**Ya-Ling Hsieh, Chia-Jung Chiang, Tsung Yu**

**Supplementary Materials**

**Table S1. Comparison of demographic and clinical characteristics between participants included and excluded from the analysis**

|  | Included  (N = 8396) | | Excluded  (N = 893) | |  |
| --- | --- | --- | --- | --- | --- |
| Variable | M or n | SD or % | M or n | SD or % | *SMD* |
| Numerical variable |  |  |  |  |  |
| Maternal age (year) | 27.0 | 5.6 | 25.7 | 6.2 | *0.222* |
| Body mass index | 26.3 | 6.3 | 27.0 | 6.7 | *-0.100* |
| Income as % of FPL | 435.4 | 308.3 | 385.3 | 321.4 | *0.159* |
| Energy intake (kcal/d) | 1710.0 | 941.7 | 1928.2 | 1292.7 | *-0.193* |
| AHEI-2010 | 55.2 | 12.5 | 52.9 | 11.9 | *0.190* |
| EPDS score | 5.7 | 4.2 | 6.6 | 4.7 | *-0.190* |
| STAI-T score | 33.8 | 8.7 | 35.2 | 9.0 | *-0.161* |
| Categorical variable |  |  |  |  |  |
| Race/ethnicity |  |  |  |  | *0.470* |
| Non-Hispanic White | 5231 | 62.3 | 364 | 40.8 |  |
| Non-Hispanic Black | 1068 | 12.7 | 180 | 20.2 |  |
| Hispanic | 1334 | 15.9 | 265 | 29.7 |  |
| Asian | 334 | 4.0 | 31 | 3.5 |  |
| Other | 429 | 5.1 | 43 | 4.8 |  |
| Education |  |  |  |  | *0.356* |
| Less than HS graduate | 635 | 7.6 | 120 | 13.4 |  |
| HS graduate or GED | 954 | 11.4 | 141 | 15.8 |  |
| Some college | 1609 | 19.2 | 221 | 24.8 |  |
| Assoc/Tech degree | 863 | 10.3 | 71 | 8.0 |  |
| Completed college | 2358 | 28.1 | 186 | 20.8 |  |
| Degree work beyond college | 1975 | 23.5 | 138 | 15.5 |  |
| Marital status |  |  |  |  | *0.411* |
| Single | 3158 | 37.6 | 500 | 56.0 |  |
| Married | 5135 | 61.2 | 358 | 40.1 |  |
| Other | 101 | 1.2 | 13 | 1.5 |  |
| Met physical activity guidelines |  |  |  |  | *0.017* |
| No | 3057 | 36.4 | 280 | 31.4 |  |
| Yes | 2836 | 33.8 | 251 | 28.1 |  |
| Smoking prior to pregnancy |  |  |  |  | *0.120* |
| No | 6918 | 82.4 | 680 | 76.2 |  |
| Yes | 1475 | 17.6 | 196 | 22.0 |  |
| Drinking prior to pregnancy |  |  |  |  | *0.103* |
| No | 1762 | 21.0 | 223 | 25.0 |  |
| Yes | 5438 | 64.8 | 545 | 61.0 |  |
| Use of vitamin |  |  |  |  | *0.689* |
| No | 958 | 11.4 | 356 | 39.9 |  |
| Yes | 7438 | 88.6 | 537 | 60.1 |  |
| Gravidity |  |  |  |  | *0.191* |
| 1 | 6290 | 74.9 | 592 | 66.3 |  |
| 2 | 1569 | 18.7 | 197 | 22.1 |  |
| >=3 | 537 | 6.4 | 94 | 10.5 |  |
| History of miscarriage |  |  |  |  | *0.061* |
| No | 7073 | 84.2 | 732 | 82.0 |  |
| Yes | 1323 | 15.8 | 161 | 18.0 |  |
| Mental health conditions |  |  |  |  | *0.020* |
| No | 6747 | 80.4 | 453 | 50.7 |  |
| Yes | 1491 | 17.8 | 95 | 10.6 |  |
| Pregestational diabetes |  |  |  |  | *0.084* |
| No | 8176 | 97.4 | 544 | 60.9 |  |
| Yes | 123 | 1.5 | 15 | 1.7 |  |
| Chronic hypertension |  |  |  |  | *0.044* |
| No | 8019 | 95.5 | 528 | 59.1 |  |
| Yes | 210 | 2.5 | 18 | 2.0 |  |

**Abbreviations:**

AHEI-2010, Alternative Healthy Eating Index 2010; Assoc/Tech degree = Associate/Technology degree; NVP = nausea and vomiting of pregnancy; EPDS = Edinburgh Postnatal Depression Scale; FPL = federal poverty level; GED = General Educational Development; HS = high school; M = mean; SD = standard deviation; SMD = standardized mean difference; STAI-T = State-Trait Anxiety Inventory-Trait Subscale

**Table S2.** **Distribution of study participants (N = 8396) by presence of medium-to-severe NVP across study visits**

| Medium-to-severe NVP at visit 1 | Medium-to-severe NVP at visit 2 | Medium-to-severe NVP at visit 3 | n (%) |
| --- | --- | --- | --- |
| No | No | No | 6849 (81.6) |
| Yes | No | No | 1182 (14.1) |
| No | Yes | No | 111 (1.3) |
| No | No | Yes | 63 (0.8) |
| Yes | Yes | No | 100 (1.2) |
| No | Yes | Yes | 45 (0.5) |
| Yes | No | Yes | 10 (0.1) |
| Yes | Yes | Yes | 36 (0.4) |

NVP = nausea and vomiting of pregnancy
